# Microbial Diversity in Bushmeat Samples Recovered from the Serengeti Ecosystem in Tanzania

**DOI:** 10.1038/s41598-019-53969-7

**Published:** 2019-12-02

**Authors:** Robab Katani, Megan A. Schilling, Beatus Lyimo, Triza Tonui, Isabella M. Cattadori, Ernest Eblate, Andimile Martin, Anna B. Estes, Teresia Buza, Dennis Rentsch, Karen W. Davenport, Blake T. Hovde, Samson Lyimo, Lydia Munuo, Francesca Stomeo, Christian Tiambo, Jessica Radzio-Basu, Fausta Mosha, Peter J. Hudson, Joram J. Buza, Vivek Kapur

**Affiliations:** 1 Applied Biological and Biosecurity Research Laboratory, Pennsylvania State University, University Park, Pennsylvania, USA; 2 The Huck Institutes of the Life Sciences, Pennsylvania State University, University Park, Pennsylvania, USA; 3 Department of Animal Science, Pennsylvania State University, University Park, Pennsylvania, USA; 4 Nelson Mandela African Institution of Science and Technology, Arusha, Tanzania; 5 Biosciences eastern and central Africa-International Livestock Research Institute (BecA-ILRI) Hub, Nairobi, Kenya; 6 Department of Biology, Pennsylvania State University, University Park, Pennsylvania, USA; 7 Tanzania Wildlife Research Institute, Arusha, Tanzania; 8 Lincoln Park Zoo, Chicago, Illinois USA; 9 Los Alamos National Laboratory, Los Alamos, New Mexico, USA; 10 Present Address: European Molecular Biology Laboratory (EMBL), Heidelberg, Germany; 11 Ministry of Health Community Development Gender Elderly and Children, Dar es Salaam, Tanzania

**Keywords:** Microbiology, Risk factors

## Abstract

Bushmeat, the meat and organs derived from wildlife species, is a common source of animal protein in the diets of those living in sub-Saharan Africa and is frequently associated with zoonotic spillover of dangerous pathogens. Given the frequent consumption of bushmeat in this region and the lack of knowledge about the microbial communities associated with this meat, the microbiome of 56 fresh and processed bushmeat samples ascertained from three districts in the Western Serengeti ecosystem in Tanzania was characterized using 16S rRNA metagenomic sequencing. The results show that the most abundant phyla present in bushmeat samples include *Firmicutes* (67.8%), *Proteobacteria* (18.4%), *Cyanobacteria* (8.9%), and *Bacteroidetes* (3.1%). Regardless of wildlife species, sample condition, season, or region, the microbiome is diverse across all samples, with no significant difference in alpha or beta diversity. The findings also suggest the presence of DNA signatures of potentially dangerous zoonotic pathogens, including those from the genus *Bacillus*, *Brucella*, *Coxiella*, and others, in bushmeat. Together, this investigation provides a better understanding of the microbiome associated with this major food source in samples collected from the Western Serengeti in Tanzania and highlights a need for future investigations on the potential health risks associated with the harvesting, trade, and consumption of bushmeat in Sub-Saharan Africa.

## Introduction

“Bushmeat” refers to the meat and organs derived from wildlife species that are frequently hunted as a food source in many countries in Africa, including Tanzania^1,2^. In Tanzania, hunting is not allowed without proper permits. However, illegal bushmeat hunting is conducted all year-round and increases during the period when migrating large herbivores, such as wildebeest (*Connochaetes taurinus*) are in close proximity to the villages contributing to the annual harvesting of ~70,000–120,000 wildebeest alone^3^. For many households, bushmeat represents an important source of animal protein, and a recent survey in regions surrounding the Greater Serengeti ecosystem suggests between 2 and 5 bushmeat meals is consumed per week per household^4^. The meat is sold as either fresh or processed (dried meat) depending on multiple factors, including the animal availability, region, taste preference, and migration season^5^. In the Serengeti ecosystem, bushmeat is primarily acquired through house-to-house sales and via middlemen traders or hunters to the end-consumers^6,7^. Notably, bushmeat consumption and trade has been increasing during recent decades due to several socio-economic factors, including growing food insecurity, demographic shifts, low cost (compared to other meat products), cultural practices, taste preferences, perceived medicinal value and prestige^8–13^.

While the majority of bushmeat is consumed locally in the regions where the wildlife is hunted, a considerable amount is illegally transported to larger cities in Africa. Bushmeat is also smuggled into the United States and Western Europe, which poses a potential global health and biosecurity risk if this meat is harbors dangerous pathogens. For instance, reports suggest that up to 5 tons of bushmeat is smuggled through a single airport in Western Europe each week^14^. These practices provide an opportunity for the transmission of dangerous pathogens and a means for the emergence of zoonotic diseases^15–17^. According to the World Health Organization, over 50% of the new infectious diseases in humans are caused by pathogens originating from animals or animal products, of which 70% have originated from wildlife^18^. Therefore, it is not surprising that recent studies have indicated that the handling and consumption of bushmeat contributes to the transmission of pathogens from animals to humans^17^.

Due to the potential global health risks associated with the consumption and transportation of bushmeat, here, we examined the microbial diversity associated with bushmeat samples harvested from the Greater Serengeti Ecosystem in Tanzania to better understand the distribution and prevalence of different bacterial communities, including possible dangers pathogens that may affect human health.

## Results

### Bushmeat samples selected for analysis

**Figure 1:**
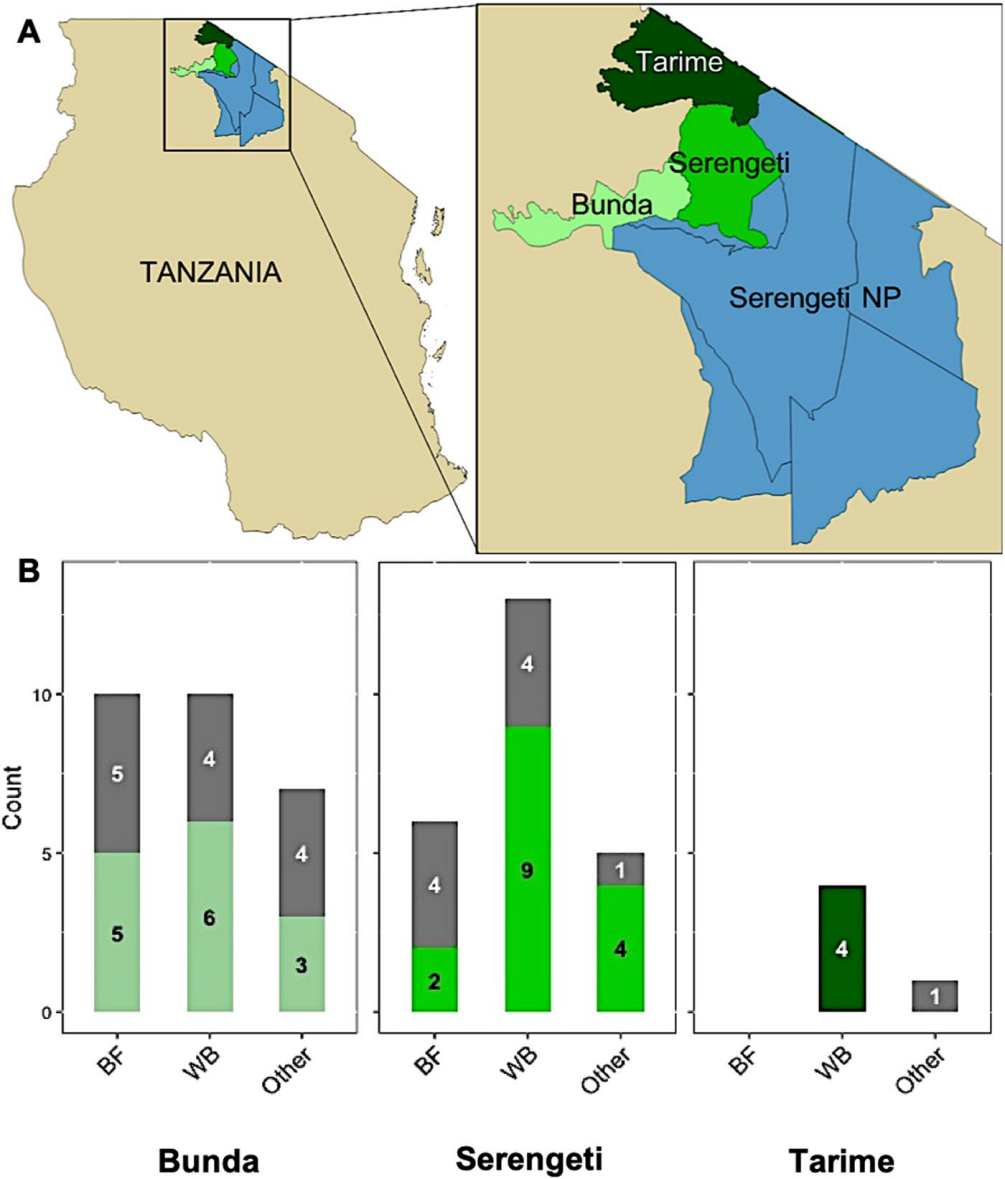
Samples from wildlife species collected from the Western Serengeti. (**A**) Samples were collected from three districts surrounding the Serengeti National Park (blue): Bunda (light green), Serengeti (medium green), Tarime (dark green). (**B**) The bar graphs represent the number of samples collected in each district (Fresh – Gray; Processed – Green shade) from wildebeest (WB), buffalo (BF), and less prevalent species (*Other*).

A total of 56 bushmeat tissue samples were harvested from representing wildlife species in the Serengeti National Park and surrounding areas. The samples represented meat from the predominant large herbivores in the region including wildebeest (*Connochaetes taurinus*, *n* = 27), buffalo (*Syncerus caffer*, *n* = 16), as well as some less common wildlife species including eland (*Tragelaphus oryx*), Thomson’s Gazelle-gazelle (*Eudorcas thomsonii*), zebra (Equus burchellii quagga), giraffe (*Giraffa camelopardalis*), rabbit (*Lepus microtis*), sheep (*Ovis aries*), topi (*Damaliscus korrigum*), porcupine (*Hystrix cristata*), and warthog (*Phacochoerus africanus*) (total *n* = 13) (Fig. 1B and Supplementary Table 1).

The samples were selected to represent fresh and processed samples from different wildlife species during both the rainy and dry seasons, and from different districts located in and around the Serengeti National Park. All samples were incidentally collected through established regional enumerator networks and teams of local wildlife veterinarians and anti-poaching units. All samples were obtained after obtaining requisite permits from the competent national authorities and in consultation with village elders and opinion makers.

### The microbial community in bushmeat

**Figure 2:**
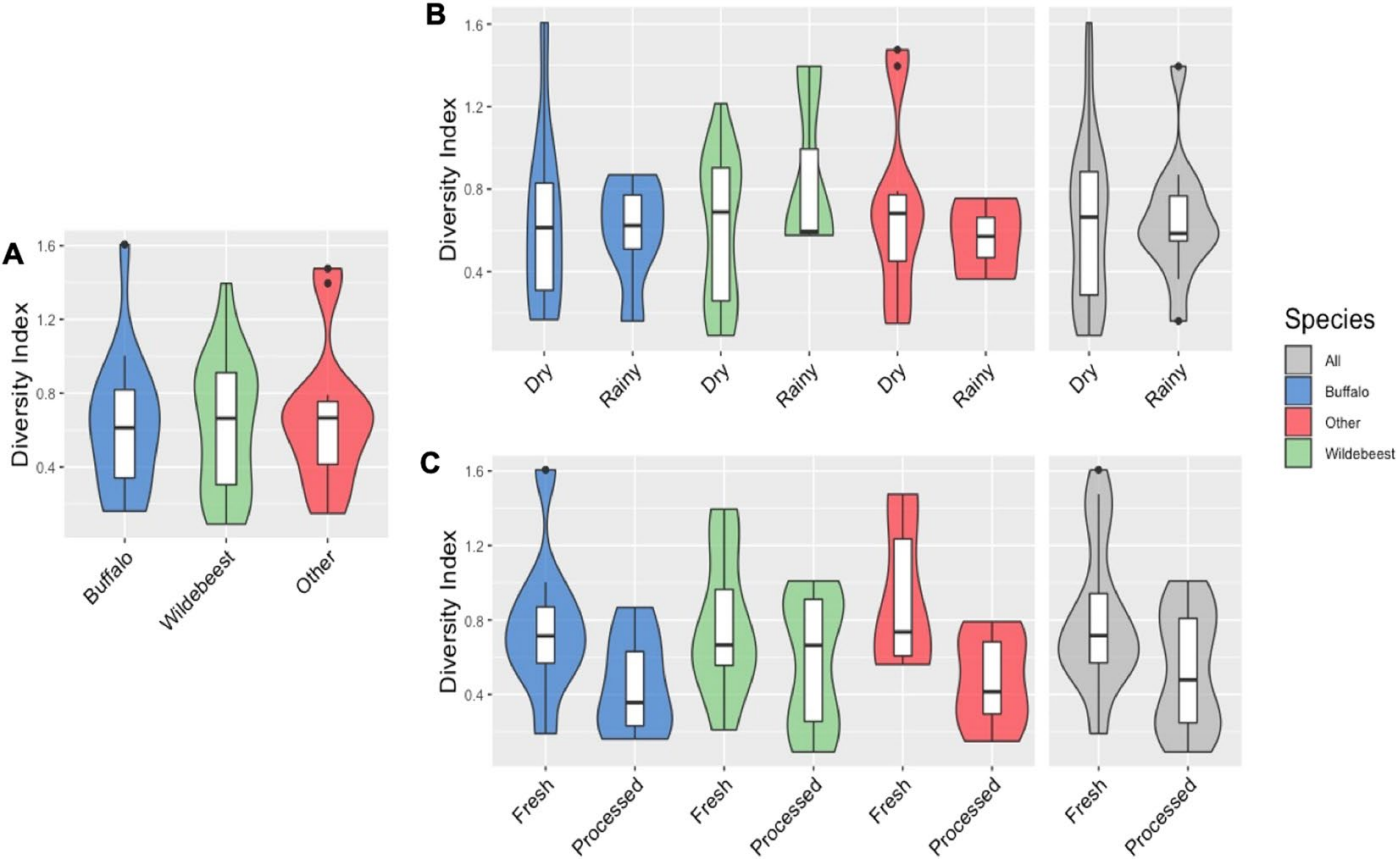
Phylum-level alpha diversity. The Shannon Diversity Index (species richness) shows no significant differences in the alpha diversity of phyla between different (**A**) wildlife species, (**B**) seasons, and (**C**) sample conditions. In the boxplot, the median is represented by the middle line, the first and third quartiles by the box and the range by the whiskers. The violin plots demonstrate the distribution of the Shannon Diversity Indices for the different variables, including buffalo (blue), wildebeest (green), Other (red). The overall Shannon Diversity Index for seasons and sample condition is represented in gray.

The results show 27 different bacterial phyla are present in the collected bushmeat samples, of which, the most abundant phyla include *Firmicutes* (67.8%), *Proteobacteria* (18.4%), *Cyanobacteria* (8.9%), and *Bacteroidetes* (3.1%). No significant differences were found in the phyla-level alpha diversity in samples collected from different species (Fig. 2A), seasons (Fig. 2B), or sample conditions (Fig. 2C). These findings were consistent at the family- and genus-levels as well (Supplementary Figs 1 and 1, respectively).

**Figure 3:**
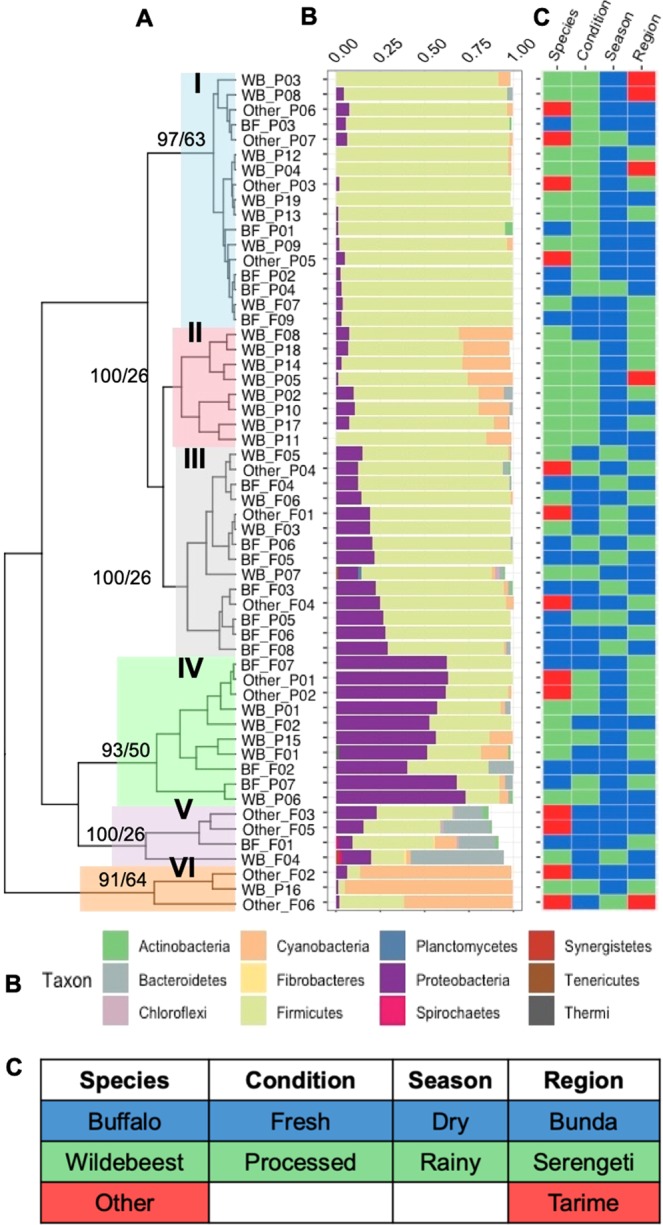
Hierarchical clustering of samples based on the relative abundance of phyla. (**A**) Hierarchical clustering grouped the samples into six clusters: C-I (Blue), C-II (Pink), C-III (Gray), and C-IV (Green), C-V (Purple), C-VI (Orange). (**B**) The stacked bar graphs represent the relative abundance of phyla in each sample. The colors are representative of the respective phyla in the legend. (**C**) The heatmap includes descriptive characteristics of each sample, including species, condition, season, and region, colored according to the legend. The approximately unbiased (AU) p-value and bootstrap probability (BP) for the respective nodes are noted (AU > 95 are statistically significant).

**Figure 4:**
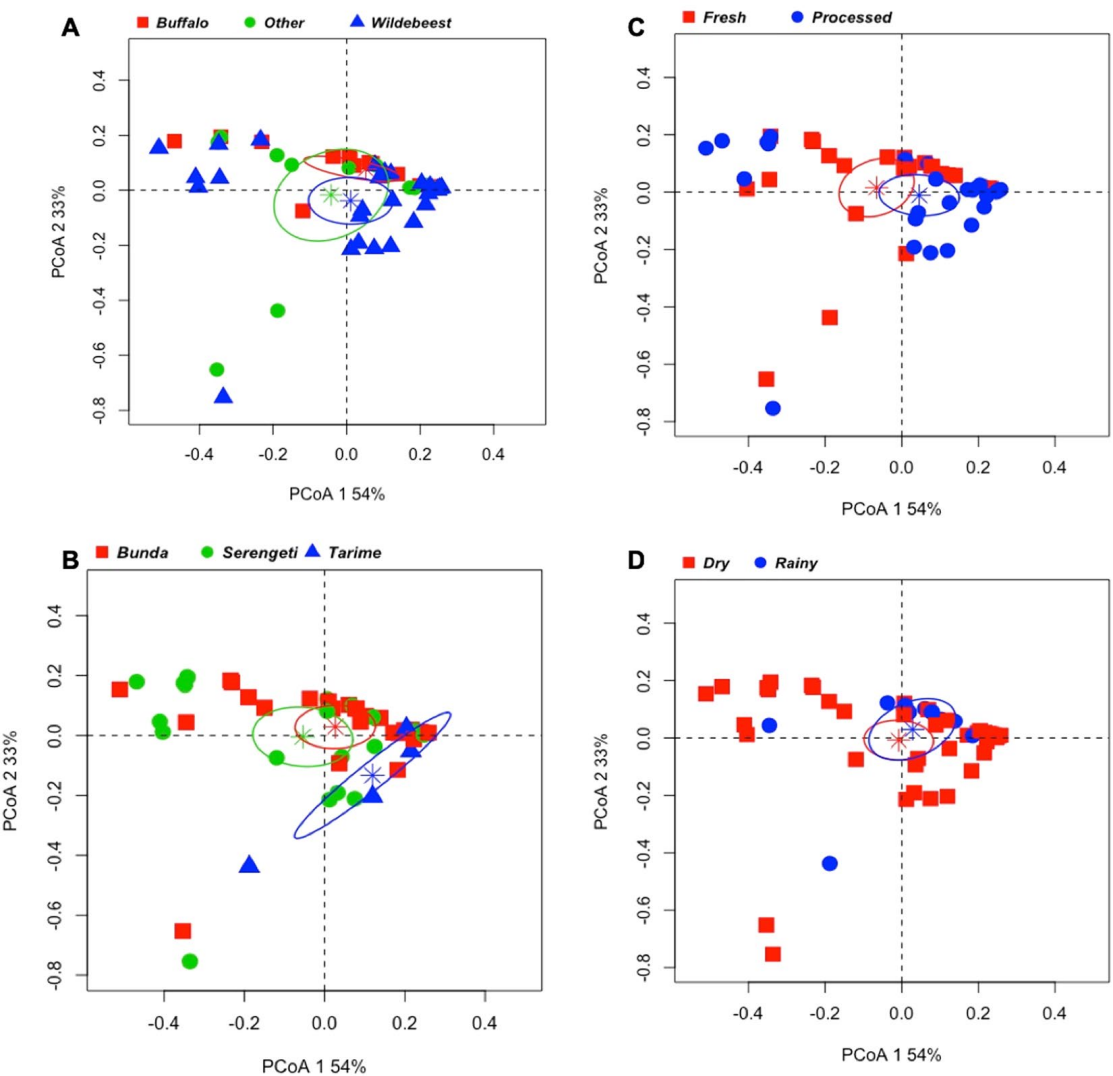
The Principal Coordinates Analysis of the microbiota at the phylum level. The beta diversity of the samples was represented in Principal Coordinate Analysis using the Bray-Curtis diversity matrix. Since there are multiple variables the four plots illustrate the same visualization of the beta diversity but colored differently according to each characteristic examined: (**A**) species, (**B**) region, (**C**) condition, and (**D**) season. In (**A**), the red square represents buffalo, the green circle represents other species, and the blue triangle represent wildebeest. In (**B**) the same symbols represent the Bunda, Serengeti, and Tarime districts, respectively. In (**C,D**), the red square represents fresh samples and dry season, respectively and the blue circle represents processed samples and rainy season, respectively. The variability explained by the first two components are represented by the percentage on each axis. The ellipses, represent the 95% confidence interval of the clustering, is represented as the respective colors in each sample series and the centers of the ellipses (or average value of the groups) are reported with the asterisk.

Hierarchical clustering based on the relative abundance of phyla of each sample was performed and approximately unbiased (AU) and bootstrap probability (BP) values of the branching patterns were computed (Fig. 3A). The samples grouped into six clusters (AU > 95), with Cluster I (*n* = 17) representing samples with the lowest diversity (an average of 95% *Firmicutes*) and included primarily processed samples collected during dry season (Fig. 3, Supplementary Table 2). Cluster II (*n* = 8) has a total of eight wildebeest samples, of which, seven are processed samples collected during dry season, mostly from the Serengeti district. These samples contain on average about 72% *Firmicutes* and 20% *Cyanobacteria* (Fig. 3, Supplementary Table 2). Clusters III (*n* = 14) and IV (*n* = 10) include samples that contain about 58% *Firmicutes*, 36% *Proteobacteria* and a small percentage of Cyanobacteria and *Bacteroidetes*. These two clusters include about half of the total samples (Fig. 3, Supplementary Table 2). Cluster V (*n* = 4) has fresh samples collected mostly during dry season. These samples have high diversity, with an average of 37% *Firmicutes*, 29% *Bacteroidetes*, and 16% *Proteobacteria* (Fig. 3, Supplementary Table 2). The smallest cluster, cluster VI, has three samples that are high in *Cyanobacteria* (80%) and low in *Proteobacteria* (3%) (Fig. 3, Supplementary Table 2). These samples include, a fresh gazelle sample collected during dry season from Bunda (*Other*_F02), a fresh warthog sample collected during rainy season from Tarime (*Other*_F06), and a processed wildebeest sample collected during dry season from Serengeti (WB_P16) (Fig. 3, Supplementary Table 2). The Principal Coordinate Analysis (PCoA) at the phylum-level was used to visualize individual and/or group differences among the samples and to describe the beta diversity in the dataset based on the relative abundance of phyla in each sample (Fig. 4). The first two principal coordinates account for 87% of the total variation (PCoA1 54% and PCoA2 33%) (Fig. 4). Although most of the clustering is not statistically significant, there is a tendency for the buffalo and wildebeest samples to cluster separately with the *other* samples category overlapping both the wildebeest and buffalo samples (Fig. 4A). The beta diversity for samples collected from each district demonstrates a significant clustering of the Tarime district compared to the Serengeti and Bunda districts, although there is a small number of samples collected from Tarime (Fig. 4B). There is no statistically significant clustering of the samples that could be explained by the sample condition or season (Fig. 4C,D). The PCoA was also performed at the family-level and the results showed significant clustering between buffalo and wildebeest samples (Supplementary Fig. 3), and also a tendency toward clustering of the samples based on sample condition and season (Supplementary Fig. 3C,D).

### Signatures of potential select agents in bushmeat

**Figure 5:**
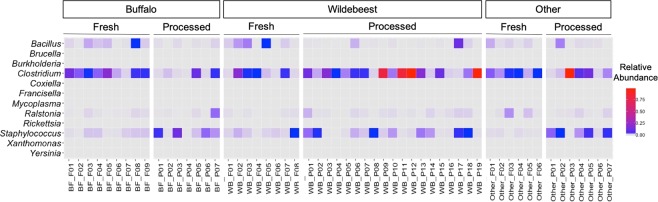
Select agents present in the bushmeat samples. The heatmap demonstrates the relative abundance of the genera of federal select agents (*Bacillus, Brucella, Burkholderia, Clostridium, Coxiella, Francisella, Mycoplasma, Ralstonia, Rickettsia, Staphylococcus, Xanthomonas, and Yersinia)* in the bushmeat samples. The relative abundance ranges from low (blue) to high (red). The gray color represents samples where the respective select agent is not present.

The data were screened for the presence of DNA signatures of potential select agents (https://www.selectagents.gov/selectagentsandtoxinslist.html), in the collected bushmeat samples. The relative abundance of the select agent genera, including *Bacillus, Brucella, Burkholderia, Clostridium, Coxiella, Francisella, Mycoplasma, Ralstonia, Rickettsia, Staphylococcus, Xanthomonas, and Yersinia* are plotted as a heatmaps (Fig. 5). The results show that all samples have the highest relative abundance of *Clostridium* (11.6%), *Staphylococcus* (0.72%), *Bacillus* (0.30%), and *Ralstonia* (0.05%). Interestingly, the analyses showed that processed wildebeest samples collected during dry season contained a high abundance of *Clostridium* species (>78%). While this data demonstrates the possible presence of select agents, it is important to note that genus-level analysis cannot confirm the presence of specific select agents in these samples. This analysis provides preliminary evidence for much needed future investigations on the presence of select agents in bushmeat samples using more directed molecular techniques to better assess public health risks associated with bushmeat consumption.

## Discussion

Although wildlife hunting in Tanzania is legal with a permit, the vast majority of bushmeat hunting is illegal and occurs in and around the major protected areas, including the Serengeti National Park. Bushmeat hunting has also been linked to increasing opportunities for pathogen transmission between wildlife and humans^16^. Hence, the goal of this study was to fill a major knowledge gap and assess the microbiome associated with bushmeat samples and the possible risk of exposure to select agents through the practices of hunting, handling, and consuming bushmeat by those living in and around Serengeti National Park.

The results of the 16S rRNA analysis indicated that at the *Firmicutes*, *Proteobacteria*, *Bacteriodetes*, and *Cyanobacteria* are the most abundant phyla across all samples in this study (Fig. 3). This is not surprising, as many common bacterial species (both pathogenic and commensal) belong to these phyla and are commonly found in the microbiomes of many other sample types. For instance, human gut microbiota has a high relative abundance of *Actinobacteria* and *Proteobacteria* during in early stages of life and *Bacteroidetes* and *Clostridium* at older ages^19^. *Firmicutes*, *Bacteroides*, and *Proteobacteria* are also found in the ceca of chicken, *Bacteroides* dominates the calf’s microbiota, and *Firmicutes*, *Bacteroidetes*, and *Proteobacteria* dominate the fecal microbiota in pig before weaning^20,21^.

Overall, the samples have high diversity irrespective of the origin of the species, season, or sample condition. This could be dependent on several factors related to the conditions of pre-processing or post-processing of the samples. Since the samples were collected along different points of the supply chain, it is difficult to speculate if these bacterial species may be endogenous to the host or have been transferred from the environment during the process of hunting, butchering, and transportation. These processes are conducted under unhygienic conditions which affects the microbiota profile of the meat as well. For instance, human skin microbiota is also mostly composed of *Proteobacteria*, *Firmicutes, Bacteriodetes*, and *Cyanobacteria*^22^. Some of the pre-capture factors that could affect the microbiota communities present in bushmeat include the movement of wildlife through different habitats and prevailing environmental conditions (which could increase susceptibility to disease), the feeding habits of the different wildlife species (grazer or browser, height at which they feed on the vegetation) and the social organization of the species (e.g. solitary or gregarious), and the interactions of wildlife with other domestic and wild species. The microbiome present in the samples could likewise be affected by the conditions during the processing of the bushmeat, including the microbiota composition of the environment and shared butchering tools^23^. For this study, the sample collection was performed on incidental basis, which reflected the variability of conditions in which bushmeat is handled and demonstrates the microbial communities present in bushmeat at the point of consumption. The samples were collected by different enumerators who might have obtained them from different sources and followed different methods of wildlife handling and sample processing prior to storing the samples for our study. Another factor that might contribute to the microbiota profile of these samples is the condition of which the samples were processed. For instance, freshly harvested bushmeat may be processed, through laying the meat on the grassy areas for weeks to dry out under the sun where these samples may come in contact with soil borne- organisms, which is consistent with our observation of the relatively higher abundance of *Cyanobacteria* (a photosynthetic microorganism) in wildebeest samples^24^.

The Shannon Diversity Index was used to compare the alpha diversity between wildlife species (Fig. 2A), season (Fig. 2B), and sample condition (Fig. 2C). The analysis was also performed at the family (Supplementary Fig. 1) and genus (Supplementary Fig. 2) levels, and the results indicated no significant differences in the alpha diversity of the samples at any taxonomic level. This could be due to the environmental factors, including all wildlife species living within the same ecosystem and sharing the same food source. All the wildlife spices in this study are herbivores and feed on grassy areas within the ecosystem, although some less selective (zebras) than others (wildebeests)^25^.

The hierarchical clustering of the samples based on the relative abundance of phyla grouped the samples into six clusters (Fig. 3). The clusters range from low diversity (Cluster I), which include mostly processed samples, to high diversity (Cluster V and VI), which include mostly fresh samples (Fig. 3B). We also observed diversity at the family-level for each cluster. For instance, cluster I includes 95% *Firmicutes* and at the family level, the samples include *Staphylococcaceae, Clostridiaceae, and Bacillales* (Fig. 3 and Supplementary Fig. 4). This is alarming as both *Streptococcaceae* and *Staphylococcaceae* belong to genera with some species that cause pathogenic infections in humans. For instance, the pathogen Methicillin-resistant *Staphylococcus aureus* is a member of *Staphylococcaceae*, and are a common cause of bacterial fermentation on food and dairy products^26^. Cluster II contained a total of eight wildebeest samples of which seven are processed sample (Fig. 3 and Supplemental Table 2). At the phylum level, the samples contain on average 72% *Firmicutes* and 20% *Cyanobacteria* (Fig. 3, Supplementary Table 1 and Supplementary Table 2). At family level, the seven processed samples contained *Streptococcaceae*, *Staphylococcaceae, Clostridiaceae* and *Leuconostocaceae*. Clusters III to VI also have high diverstiy at the family level, and include *Bacillaceae*, *Enterobacteriaceae, Peptostreptococcaceae, Moraxellacear*, and *Pseudomonadaceae* (Fig. 3, Supplemental Fig. 4 and Additional Text File 1). Although some of the bacteria in this family are commensal, some are pathogens and harmful to human. For instance, some *Enterobacteriaceae* produce endotoxins that could cause cells lysis in the host leading to vasodilatory response due to the systemic inflammation^27^.

The beta diversity indicated an overall high diversity amongst all samples. Interestingly, however, at the phylum level, we observed significant clustering of samples from the Tarime district in comparison with samples collected from the Bunda and Serengeti districts. There are five samples collected from the Tarime district, of which four are processed wildebeest samples collected during dry season and belong to clusters I and II where the samples represent phyla with the least diversity (Fig. 4B). Although this is a small sample size of wildebeest from Tarime, this observation could suggest temporal clustering of wildebeest’s samples where they may have shared resources or were butchered and processed in proximity to each other.

There is also a tendency of clustering of buffalo and wildebeest samples in the PCoA plot with the *Other* category overlap both (Fig. 4A). Interestingly, at the family-level, the results from PCoA analysis indicate significant clustering between buffalo and wildebeest samples (Supplementary Fig. 3A). This clustering might reflect the endogenous nature of microbiota in each wildlife species; however, other factors might also be contributing to the clustering. For instance, only processed wildebeest samples were collected from Tarime, while all buffalo samples (fresh and processed) and fresh wildebeest samples were collected from both Bunda and Serengeti districts. Therefore, the clustering of wildebeest and buffalo samples may be related to handling, transporting or selling of the meat in different markets in the three districts. This might be one contributing factors, for the significant clustering in samples collected from the Tarime district. There could be other environmental factors affecting the clustering including the path of the annual migration of wildebeests. As the wildebeests migrate through Serengeti, the different soil, vegetation, water sources and other factors, including distance traveled from where bushmeat was acquired to where it is sold, could play roles in the microbiota composition of the wildebeest samples^28,29^. At the family- level, we observe a tendency toward clustering of the samples based on both season and condition (Supplementary Fig. 3C,D). In Tanzania, several methods of bushmeat processing are practiced, including addition of high salt, smoking, semi- boiling, or sun-drying (or any combinations) and this may contribute to the diversity in the processed samples and highlights the need future investigations^30^.

Since there is a potential human health risk associated with bushmeat consumption, the presence of select agents was assessed. The presence of certain phyla, including the high relative abundance of *Firmicutes*, that contain both commensal bacteria and pathogenic bacteria pose a potential health risk associated with these samples and strongly argues for further investigation of bushmeat as a global health concern. For instance, *Staphylococcus aureus* belongs to the phylum of *Firmicutes* and is associated with pneumonia, meningitis, and arthritis in humans^31^. Also, several species of *Clostridium* are pathogenic to humans, including *C. botulism* which causes Botulism, *C. tetani* which causes tetanus, and *P. copri* that has been associated with the onset of rheumatoid arthritis^32,33^. Therefore, to understand the risks associated with the handling and consumption of bushmeat, we further examined the microbiome content at the genus-level for the federal select agents. The analysis indicated that both fresh and processed samples from all wildlife species samples contained high levels of *Clostridium* and *Staphylococcus* (Fig. 5). The abundance of *Staphylococcus* is higher in processed samples than fresh samples across all species (Fig. 5). In addition, *Bacillus* is also prominent across many of the collected samples. This pathogen could be transmitted from animals to human, particularly through direct contact with infected animals or animal tissues. This is specifically noteworthy considering many species of *Bacillus* are pathogenic and have led to widespread outbreaks in the study region^34^. The analysis detected Yersinia, which includes the select agent *Y. pestis*, a well-known zoonotic pathogen that causes plague in humans. Although we were only able to examine the select agents at the genus level in this study, these results suggest the need for future studies to investigate bushmeat using more targeted species-level analysis of the select agents.

The observed presence of signatures of select agents in bushmeat samples in this study suggests potential for human exposures to new and emerging zoonotic pathogens from this important food source in Tanzania. To our knowledge, this is the first report of the microbiome of bushmeat collected from a savannah ecosystem. The overall results indicate a possible risk of exposure to  zoonotic pathogens associated with bushmeat hunting, butchering, handling, and consumption. Further molecular analyses including deep sequencing need to be performed in order to assess the true risk associated with spillover of specific especially dangerous pathogens from bushmeat.

## Methods

### Study area-the Greater Serengeti ecosystem (Serengeti)

The ~25,000 km^2^ Greater Serengeti Ecosystem is located in northern Tanzania and southern Kenya^35^. The ecosystem in Tanzania consists of several protected areas including the Serengeti National Park (14,760 km^2^, between 1° and 3°30S and 34° and 36°E) and adjacent game reserves and game-controlled areas. There are 27 species of large mammal herbivores inhabiting the Serengeti ecosystem^35^, of which wildebeest, zebra, Thomson’s gazelle, buffalo, topi, and eland are the most abundant grazing species^25,36^. There are also close to 1.5 million white-bearded wildebeest, 250,000 plains zebra, and 500,000 Thomson’s gazelle that seasonally migrate between the Serengeti ecosystem and the Maasai Mara in southern Kenya^4^. The climate of the Serengeti is considered subtropical, with a bimodal rainfall pattern consisting of two rainy and two dry seasons. There is a “short rainy season” from November to December and a “long rainy season” from March to May, with the average annual rainfall varies from 600 mm to 1100 mm per year, depending on the region^35^. To ensure a proper temporal representation of the presence of microbes in bushmeat, samples were collected throughout the year, including during both rainy and dry seasons.

Spatially, samples were collected from the Bunda, Serengeti, and Tarime districts located in the Western part of the Serengeti ecosystem (Fig. 1A). The Bunda district (coordinates 02°00′S 33°50′E) is the Western-most District adjacent to Serengeti National Park, bordering the shores of Lake Victoria. The Tarime district (coordinates 01° 20′S 34°23′E) is bordered from the north by Kenya; the Mara River delineates the boundary between Tarime and Serengeti districts. The Serengeti district (01°50′S 34°40′E) includes much of the Ikorongo and Grumeti Game Reserves, Ikona WMA and part of Serengeti National Park. The Serengeti National Park and the areas surrounding the game reserves are hotspots for human-wildlife interactions. The game reserves serve as buffer zones between local communities and the national park, and where the wildlife hunting occurs^6^. Most of the communities where the samples were collected from are adjacent to the game reserves. The human population densities vary amongst the districts; Bunda District has the highest (91.3 persons per km^2^) followed by Serengeti District (16.1 persons per km^2^)^35^.

### Bushmeat sample ascertainment

Sample collection occurred between September 2016 and March 2017. Bushmeat samples were collected and classified as either “fresh” or “processed”. Fresh samples were those taken from raw, uncured bushmeat, while processed samples were those which were dried by the hunters, a preservative measure to allow them to last longer. Sample identification sheets were completed for all collected samples and the metadata, including information regarding sample condition, the collection site (villages using GPS coordinates), seasonality, and date of collection were recorded (Fig. 1B and Supplementary Table 1).

Approximately 250–500 g of muscle Samples were collected in sterile 50 ml Falcon conical centrifuge tubes (Thermo Fisher Scientific, Waltham, Massachusetts) and stored in −20 °C vehicle freezers until arrival at Serengeti wildlife veterinary laboratory where they were transferred to −20 °C solar freezers. For larger tissues, double zip lock freezer bags containing non-toxic Silica Gel desiccant moisture absorber/dehumidifier were used to store the samples. The samples were then transported to the Nelson Mandela African Institute of Science and Technology (NM-AIST) in Arusha via vehicle freezers, where they were stored at −80 °C until processing. Cold chain maintenance was ensured through temperate trackers included with the samples throughout the entire process.

This study and all the methods were approved and performed in accordance with the relevant guidelines and regulations with permits from the appropriate institutions, including the Tanzania Wildlife Research Institute (TAWIRI; permit number TWRI/RS-331/VOL.II/2013/58 and TWRI/RS-331/Volume II/2013/88), Tanzania National Parks Authority (TANAPA; permit number TNP/HQ/C.10/13), Ngorongoro Conservation Area Authority (NCAA;  permit number NCAA/D/240/Volume XXVIII/54) and Tanzania Wildlife Authority; permit number CB.517/519/01/14.

### Nucleic acid extraction

From each sample, three small sections, weighing approximately 120 mg total, were dissected from both superficial and deep muscle tissue, using a sterile, disposable Rapid Core punch (World Precision Instruments, Sarasota, FL) or sterile disposable safety scalpels (VWR, Bridgeport, NJ) and placed in the MagMAX™ Lysis/Binding Solution Concentrate buffer (Thermo Fisher Scientific, Waltham, Massachusetts). Homogenization was performed using the company’s recommended procedure with slight modifications. In brief, using the Bead Ruptor 24 Bead Mill Homogenizer (Omni International, Kennesaw, GA), fresh samples were processed for 45 second at 5.5 m/s using 2.3 MM zirconia beads (BioSpec Products, Bartlesville, OK) in the MagMAX™ Lysis/Binding buffer. Processed samples were pre-soaked in UltraPure DNase/RNase-Free Distilled Water (Thermo Fisher Scientific, Grand Island, NY) at 4 °C overnight, followed by beating for three 30 second intervals at 5.5 m/s with 2.3 MM zirconia beads in the MagMAX™ Lysis/Binding buffer. Nucleic acid extractions were performed using MagMAX™ 96 DNA Multi-Sample Kit (Thermo Fisher Scientific, Grand Island, NY) and KingFisher Flex automated DNA purification system (Thermo Fisher Scientific, Grand Island, NY) from tissue samples per manufacturer’s instructions with minor modifications. If the yield and purity of extractions were not adequate for a specific sample, such as the DNA concentration was less than 4 ng/μl and/or if the quality of the amplicons analyzed by the Bioanalyzer did not pass the quality control, DNA was extracted manually using DNeasy PowerSoil Kit (Qiagen, Hilden, Germany) per manufacturer’s instructions. Extracted DNA was quantified using Qubit™ 3.0 Fluorometer (Thermo Fisher scientific, Grand Island, NY). To ensure purified DNA was of high-quality, DNA was also visualized through agarose gel electrophoresis.

### Microbiome 16S rRNA sequencing

The purified DNA was transported on dry ice to the Biosciences eastern and central Africa-International Livestock Research Institute (BecA-ILRI) Hub in Nairobi, Kenya for the microbiome analysis. Using previously published primers^37,38^, the V4 hyper-variable region of the 16S rRNA gene was amplified from the DNA extracts during the first PCR step using the universal 515 F forward primer (5′ GTGCCAGCMGCCGCGGTAA 3′) and uniquely barcoded 806 R reverse primers (5′ GGACTACHVGGGTWTCTAAT 3′) for each sample. The PCR was performed using 25 μl Bioneer premix (Bioneer, Alameda, CA). The premix was complemented with 1.5 μl each of 10 μM forward and reverse primers (Bioneer, Alameda, CA 10 μl Nuclease-free water (Ambion, Life Technologies, Waltham, MA) and 5 μl genomic DNA in a final volume of 50 μl. The PCR reaction was carried out on a Thermal Cycler (Life Technologies, Carlsbad, CA) as follows: an initial denaturation step (95 °C for 1 min), 35 cycles of amplification (95 °C for 30 sec, 60 °C for 30 sec and 72 °C for 30 sec) and a final elongation step at 72 °C for 5 min. Amplicons were then purified using the QIAquick PCR purification kit (Qiagen, USA) following the manufacturer’s instructions. The concentration of the purified amplicons was analyzed using Qubit dsDNA BR Assay kit (ThermoFisher Scientific, Waltham, MA).

Sample multiplexing was performed using a unique reverse primer per sample^40^, which was added during the PCR step. The quality of a set of amplicons (per sequencing run) was tested using Agilent DNA 7500 chips using the Bioanalyzer 2100 (Agilent Technologies, Santa Clara, CA, USA). 40 ng of each sample library was pooled in order to normalize the libraries. The final pool was denatured (NaOH 0.2 N) and diluted to 12 pM The PhiX Control V3 was added to the pool at 15% of the final concentration as described in the Illumina procedure for sequencing of low diversity libraries. Six hundred μl of this treated pool mixture was loaded onto the Illumina MiSeq cartridge according to the manufacturer’s instructions and sequenced on the Illumina MiSeq System generating 2 × 151 bp Paired-End Reads.

### Bioinformatics for microbiota composition

To produce a high-quality data set, microbiome analysis was performed using an open source platform, Empowering the Development of Genomics Expertise (EDGE)^39^. Analysis of demultiplexed paired-end 16S rRNA gene reads was completed with QIIME (v1.9.1)^40^ incorporated into the EDGE Bioinformatics platform (v2.3.0)^39^. EDGE implementation is based on QIIME v1.9.1 and includes demultiplexing and quality filtering, operational taxonomic unit (OTU) clustering, taxonomic assignment, phylogenetic reconstruction, diversity analyses and visualizations of each of these analyses. QIIME started the pre-processing step with 18,452,934 paired-end reads (in FASTQ format) by removing reads with a PHRED quality score lower than Q20. Data were further filtered to remove reads with more than one ambiguous base. If less than 50% of a read contained consecutive high-quality bases, the read was discarded. The forward and reverse reads were merged before analysis. A 94% nucleotide identity sequence clustering threshold (approximately genus level) was used to generate OTUs.

The representative sequence for each OTU was then queried against the Greengenes database^41^ for taxonomic classification assignment. The depth filter was set to 1000 reads – any barcoded sample that had less than 1000 assembled reads did not undergo taxonomic classification or further alpha- and beta-diversity analyses. Next, the integrated phylotype command generated the consensus taxonomy using phylotype- based approach, where taxonomy-linked sequences were assigned to OTUs based on similarity.

### Statistical analysis

To calculate the potential for sample size biases in the microbiota sequencing, a rarefaction analysis was conducted where the fraction of taxa was captured for each sample^42^. Results show that we were able to consistently identify all the detectable OTUs present in every sample with a minimum reading depth of 6,470 sequences per sample. This suggests that the variation of the microbiota among samples is not likely caused by low sequence coverage (Supplementary Fig. 5) and that further sequencing would not detect additional genera.

The microbiome alpha diversity (species richness) in relation to the different characteristics of the samples (fresh versus processed; dry versus rainy season; buffalo versus wildebeest, versus *other*) was calculated using the Shannon Diversity Index (R package: Vegan) at the phylum-, family-, and genus-level^43^. Statistical analysis between the groups was performed using a t-test in R^44^. Hierarchical clustering of the samples based on relative abundance of phyla was performed using the R, pvclust, with 1000 bootstrapped replications of the clusters^45^. To examine the beta diversity of the samples, PCoA was performed using the Bray-Curtis Matrix at the phylum level using the R package, Vegan^43^. Groups were clustered drawing ellipses estimated as the 95% confidence region of the joint distribution of the two components considered. Statistical analysis of the PCoA data was performed using the R package, Vegan^43^ (Supplementary Tables 3 and 4).

### Accession codes

The data is publicly available at: https://trace.ncbi.nlm.nih.gov/Traces/sra/?study=SRP151593.

## Supplementary information

Additional Text file 1

Supplementary Figures

Supplementary Tables

Supplementary Table 1

Supplementary Table 2

Supplementary Table 3

Supplementary Table 4
