# Supplementary material for: Microbial Diversity in Bushmeat Samples Recovered from the Serengeti Ecosystem in Tanzania
Source: Sci Rep. 2019 Dec 2;9:18086. doi: 10.1038/s41598-019-53969-7 (PMC6888819; doi:10.1038/s41598-019-53969-7)
Supplement: Supplementary file 1 — Additional Text file 1 [file 41598_2019_53969_MOESM1_ESM.docx]

**Additional Text File 1. In depth description of each cluster from Figure 3.**

**Microbial Diversity in Bushmeat Samples Recovered from the Serengeti Ecosystem in Tanzania**

**Robab Katani^1,2^, Megan A. Schilling^2,3^, Beatus Lyimo^4^, Triza Tonui^5^, Isabella M. Cattadori^2,6^, Ernest Eblate^4,7^, Andimile Martin^4^, Anna B. Estes^2,4^, Teresia Buza^2^, Dennis Rentsch^8^, Karen W. Davenport^9^, Blake T. Hovde^9^, Samson Lyimo^4^, Lydia Munuo^4^, Francesca Stomeo^5^, Christian Tiambo^5^, Jessica Radzio-Basu^1,2^, Fausta Mosha^10^, Peter J. Hudson^1,2,7^, Joram J. Buza^4^ and Vivek Kapur^1,2,3,4*^**

1Applied Biological and Biosecurity Research Laboratory, Pennsylvania State University, University Park, Pennsylvania, USA;

2The Huck Institutes of the Life Sciences, Pennsylvania State University, University Park, Pennsylvania, USA;

3Department of Animal Science, Pennsylvania State University, University Park,

Pennsylvania, USA;

4Nelson Mandela African Institution of Science and Technology, Arusha, Tanzania; 5Biosciences eastern and central Africa-International Livestock Research Institute (BecA-ILRI) Hub, Nairobi, Kenya (Currently at the European Molecular Biology Laboratory (EMBL), Heidelberg, Germany);

6Department of Biology, Pennsylvania State University, University Park; ^7^Tanzania

Wildlife Research Institute, Arusha, Tanzania;

8Lincoln Park Zoo, Chicago, Illinois, USA;

9Los Alamos National Laboratory, Los Alamos, New Mexico, USA;

10Ministry of Health Community Development Gender Elderly and Children, Dar es Salaam, Tanzania.

Within cluster I samples, we observed a range of different families within the Firmicutes phylum (Supplemental Figure 4). For instance, there are five buffalo samples in this cluster of which four are processed samples collected from Bunda district. One sample (BF_P01) contains 55% *Staphylococcaceae*, two samples (BF_P02 and BF_P04) are abundant in *Peptostreptococcaceae* (>90%), and sample BF_P03 is high in *Bacillales* (>60%) (Figures 3, and Supplemental Figure 4A). Cluster I also include eight wildebeest samples, where seven of them are processed samples collected during dry season and from all three districts. Similar to the buffalo samples in this cluster, the wildebeest samples are high in the Firmicutes at phylum level, and diverse at the family level. For instance, WB_P03, WB_P09, WB_P12, and WB_P19 all have high level of *Clostridiaceae*, whereas WB_P04 and WB_P07 are high in the *Peptostreptococcaceae* (Figures 3 and Supplemental Figure 4B). Included in this cluster are also four processed samples belonging to the Other species group, an eland (Other_P03), a porcupine (Other_P05), a topi (Other_P06), and a zebra (Other_P07) that were collected during both rainy and dry season and from Bunda and Serengeti districts, all have high level of Firmicutes at the phylum level, and differ at the family level, similar to buffalo and wildebeest samples, indicating lower diversity amongst this cluster at the phylum level and higher diversity at the family level (Figures 3 and Supplemental Figure 4C).

Clusters III and IV contain 10 buffalo samples (seven fresh, three processed), nine wildebeest samples (five fresh, four processed), and five samples from the Other species. Cluster IV includes fresh and processed buffalo, wildebeest, and *Other* samples collected during dry season and from Serengeti district (Figure 3, Supplemental table S2). Well diverged at the family level the cluster includes, *Bacillaceae*, *Enterobacteriaceae*, *Peptostreptococcaceae*, *Moraxellacear*, *and Pseudomonadaceae*.

Cluster V includes all fresh samples collected mostly during dry season, and the samples are highly diverse at the phylum level. Samples belonging to this cluster are from different species and collected from both seasons. For instance, there are two *Other* samples, rabbit (Other_F03) and a topi (Other_ F05), both collected fresh during dry season, and from Bunda district and at phylum level are high in Proteobacteria and Firmicutes, respectively, and a wildebeest sample (WB_F04) also collected fresh during rainy season from Bunda district, and at the phylum level is high in Bacteroidetes (Figure 3, Supplemental Table 1 and 2, and Supplemental Figure 4).

Samples belonging to this cluster includes, a fresh gazelle sample collected during dry season from Bunda (Other_F02), a fresh warthog sample collected during rainy season from Tarime (Other_F06), and a processed wildebeest sample collected during dry season from Serengeti (WB_P16). The last cluster, VI includes three samples that are high in Cyanobacteria and Chloroplast at the class level (Figure 3, Supplemental Table 2).
