# Supplementary material for: Microbial Diversity in Bushmeat Samples Recovered from the Serengeti Ecosystem in Tanzania
Source: Sci Rep. 2019 Dec 2;9:18086. doi: 10.1038/s41598-019-53969-7 (PMC6888819; doi:10.1038/s41598-019-53969-7)
Supplement: Supplementary file 2 — Supplementary Figures [file 41598_2019_53969_MOESM2_ESM.docx]

**Microbial Diversity in Bushmeat Samples Recovered from the Serengeti Ecosystem in Tanzania**

**Robab Katani^1,2^, Megan A. Schilling^2,3^, Beatus Lyimo^4^, Triza Tonui^5^, Isabella M. Cattadori^2,6^, Ernest Eblate^4,7^, Andimile Martin^4^, Anna B. Estes^2,4^, Teresia Buza^2^, Dennis Rentsch^8^, Karen W. Davenport^9^, Blake T. Hovde^9^, Samson Lyimo^4^, Lydia Munuo^4^, Francesca Stomeo^5^, Christian Tiambo^5^, Jessica Radzio-Basu^1,2^, Fausta Mosha^10^, Peter J. Hudson^1,2,7^, Joram J. Buza^4^ and Vivek Kapur^1,2,3,4*^**

^1^Applied Biological and Biosecurity Research Laboratory, Pennsylvania State University, University Park, Pennsylvania, USA;

^2^The Huck Institutes of the Life Sciences, Pennsylvania State University, University Park, Pennsylvania, USA;

^3^Department of Animal Science, Pennsylvania State University, University Park, Pennsylvania, USA;

^4^Nelson Mandela African Institution of Science and Technology, Arusha, Tanzania;

^5^Biosciences eastern and central Africa-International Livestock Research Institute (BecA-ILRI) Hub, Nairobi, Kenya (Currently at the European Molecular Biology Laboratory (EMBL), Heidelberg, Germany);

^6^Department of Biology, Pennsylvania State University, University Park; ^7^Tanzania Wildlife Research Institute, Arusha, Tanzania;

^8^Lincoln Park Zoo, Chicago, Illinois, USA;

^9^Los Alamos National Laboratory, Los Alamos, New Mexico, USA;

^10^Ministry of Health Community Development Gender Elderly and Children, Dar es Salaam Tanzania.

**Supplementary Document- Supplementary Figures**


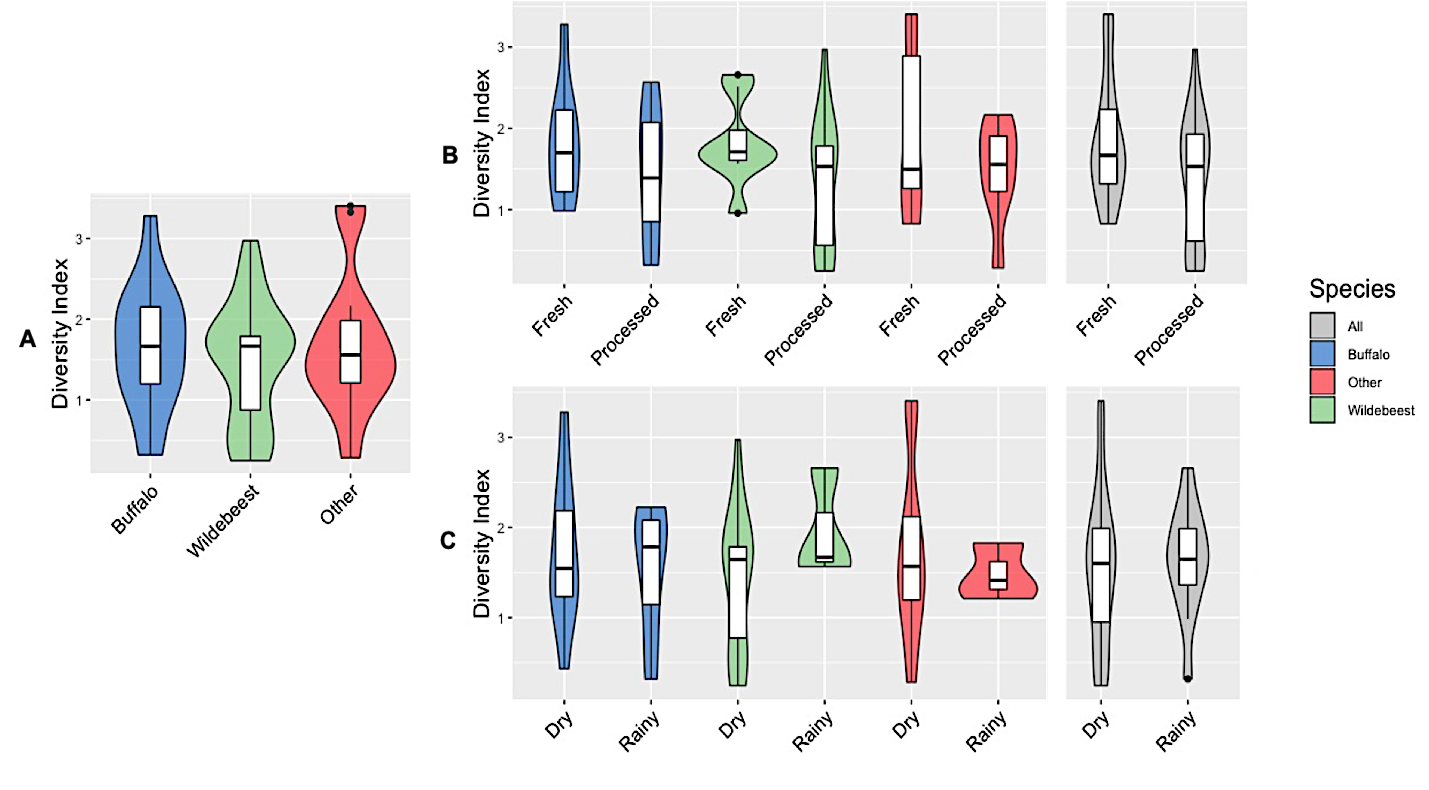


**Supplementary Figure 1. Family-Level Shannon Diversity Index (Alpha diversity**). The Shannon Diversity Index (species richness) shows no significant differences in the

alpha diversity of phyla between different (A) wildlife species, (B) seasons, and (C) sample conditions. In the boxplot, the median is represented by the middle line, the first and third quartiles by the box and the range by the whiskers. The violin plots demonstrate the

distribution of the Shannon Diversity Indices for the different variables, including buffalo (blue), wildebeest (green), Other (red). The overall Shannon Diversity Index for seasons and sample condition is represented in gray.

**Supplementary Figure 2. Genus-Level Shannon Diversity Index (Alpha diversity**). The Shannon Diversity Index (species richness) shows no significant differences in the

alpha diversity of phyla between different (A) wildlife species, (B) seasons, and (C) sample conditions. In the boxplot, the median is represented by the middle line, the first and third quartiles by the box and the range by the whiskers. The violin plots demonstrate the

distribution of the Shannon Diversity Indices for the different variables, including buffalo (blue), wildebeest (green), Other (red). The overall Shannon Diversity Index for seasons and sample condition is represented in gray.


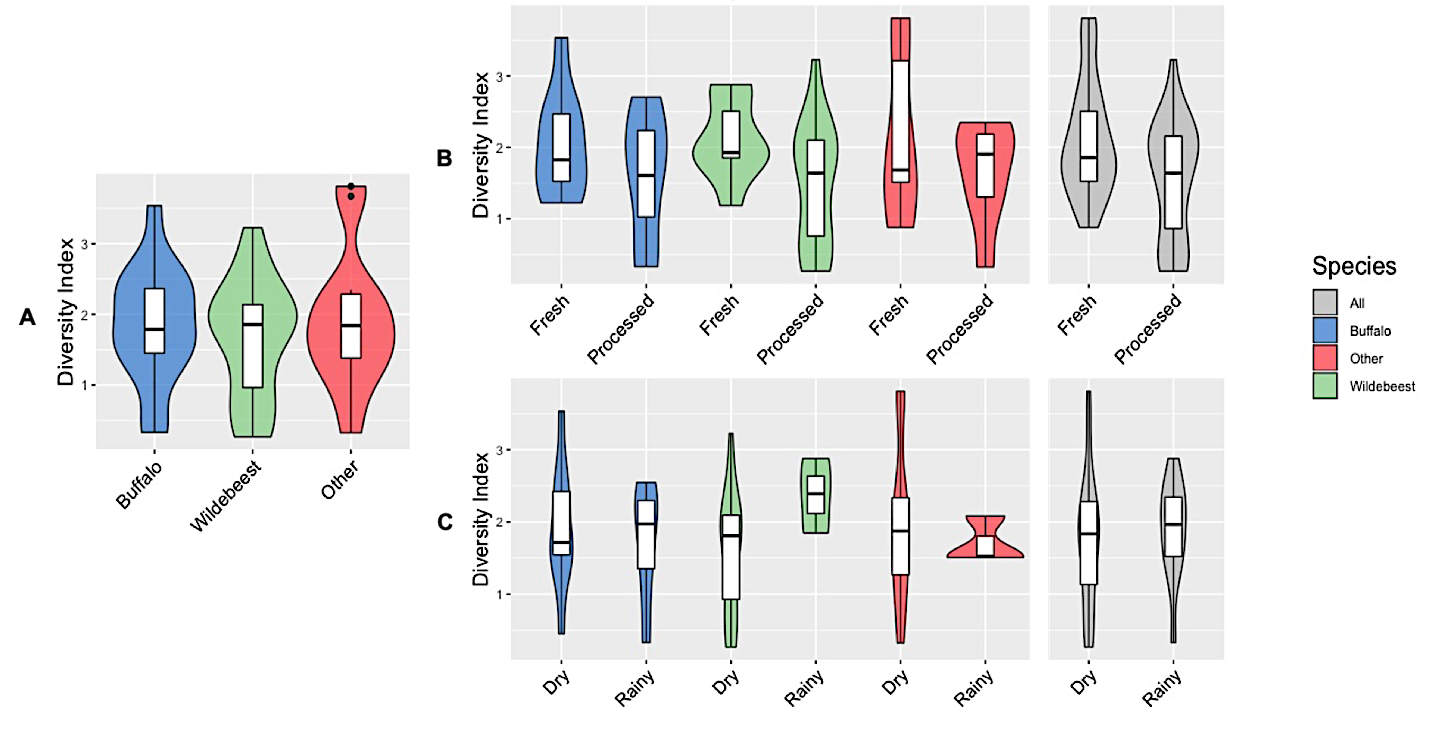


**Supplemental Figure 3. The Principal Coordinates Analysis of the microbiota at the family level.** The beta diversity of the samples was represented in Principal Coordinate Analysis using the Bray-Curtis diversity matrix. Since there are multiple variables the four plots illustrate the same visualization of the beta diversity but colored differently according to each characteristic examined: (A) species, (B) region, (C) condition, and (D) season. In (A), the red square represents buffalo, the green circle represents other species, and the blue triangle represent wildebeest. In (B) the same symbols represent the Bunda, Serengeti, and Tarime districts, respectively. In (C) and (D), the red square represents fresh samples and dry season, respectively and the blue circle represents processed samples and rainy season, respectively. The variability explained by the first two components are represented by the percentage on each axis. The ellipses, represent the 95% confidence interval of the clustering, is represented as the respective colors in each sample series and the centers of the ellipses (or average value of the groups) are reported with the asterisk.


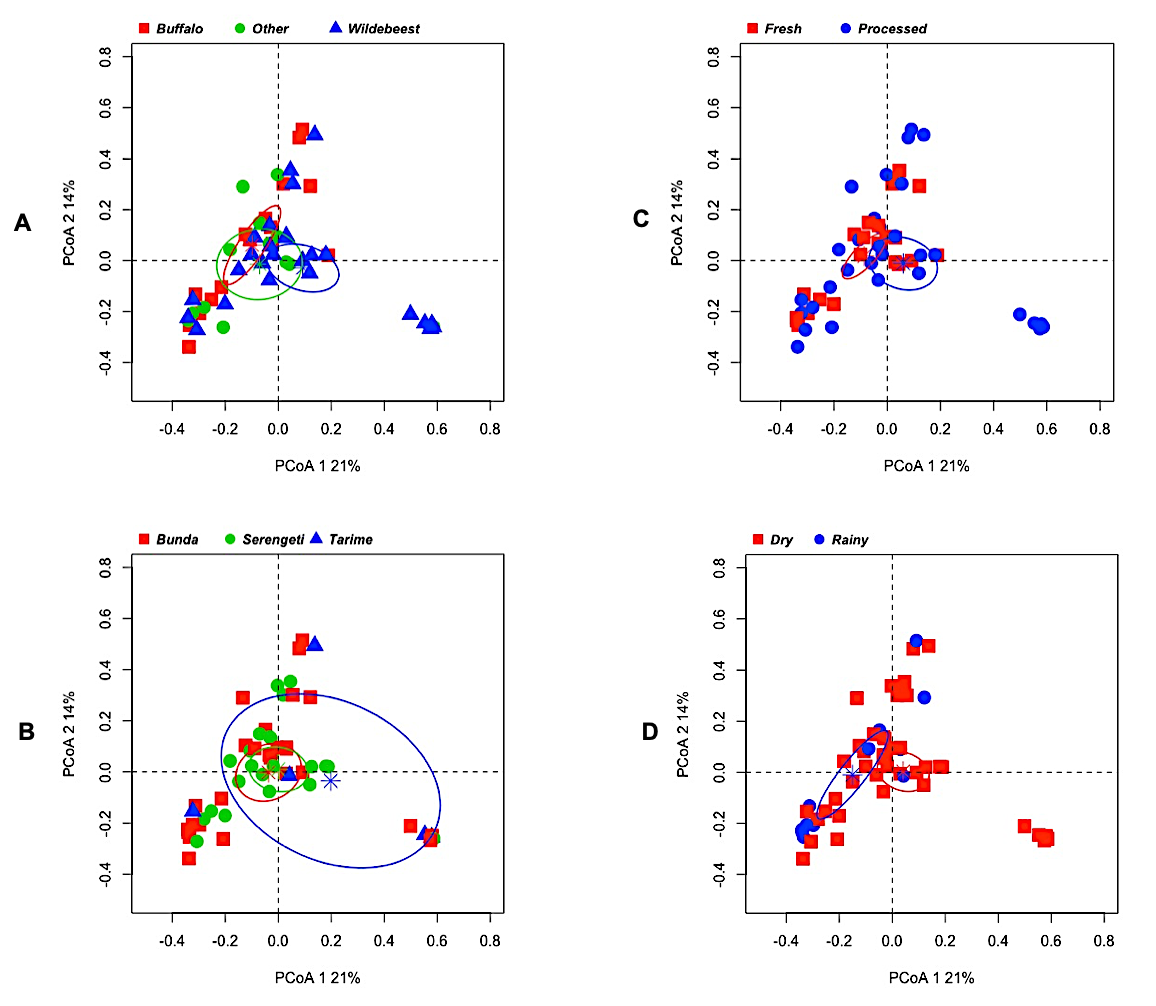


**Supplementary Figure 4. The bar charts show relative abundance of all samples at the family-level.**  Relative abundances are shown in buffalo (A), wildebeest (B), and other species (C).


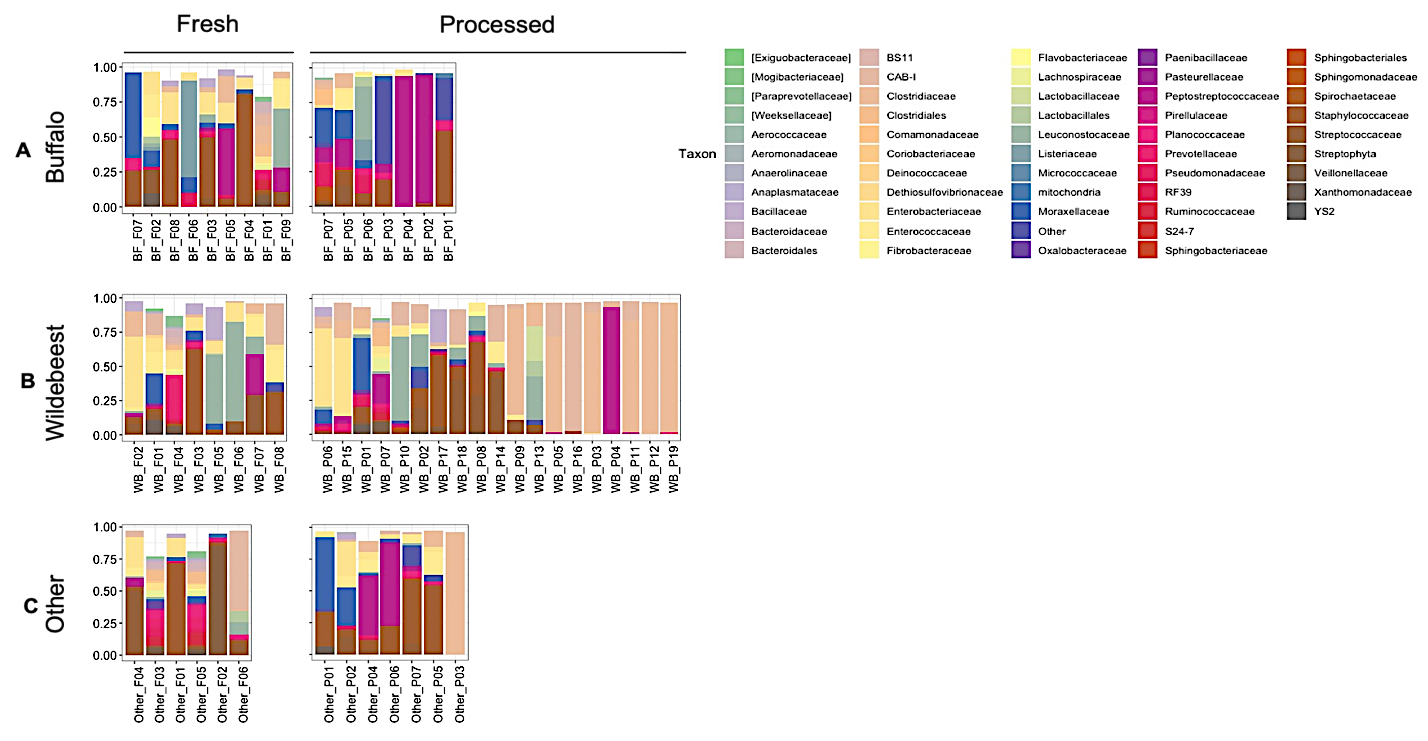


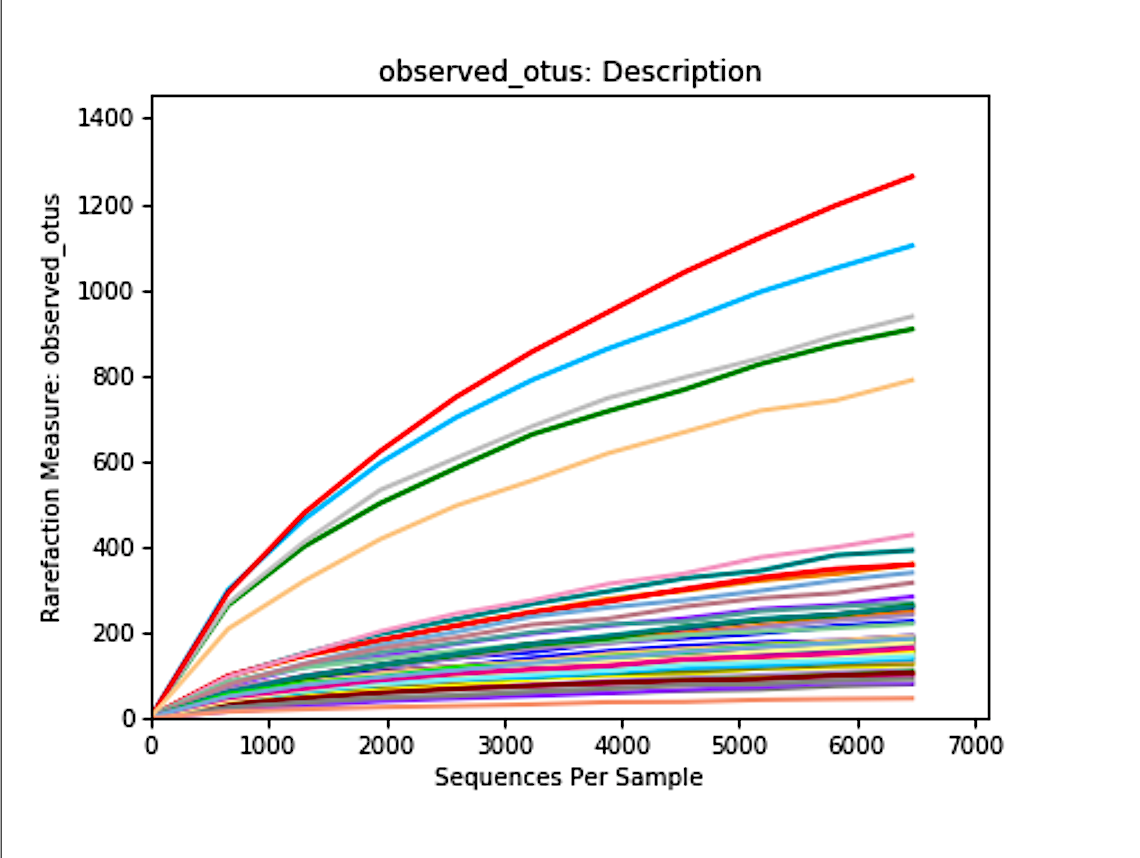


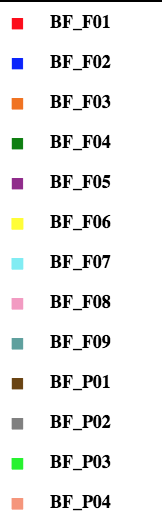

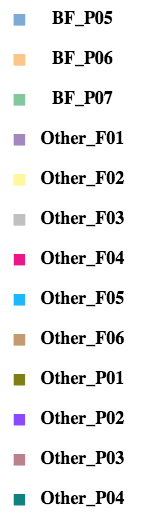

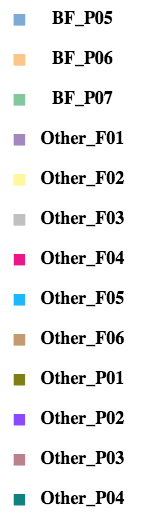

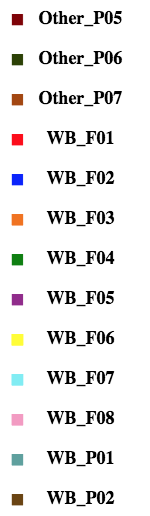

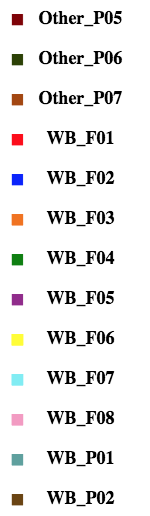

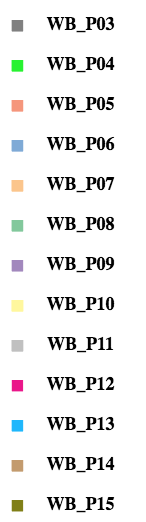

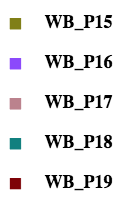


**Legend**

**Supplementary Figure 5. Rarefaction Analysis.** Each line represents the rarefaction curve for each specific sample (some samples might overlap with each other). These curves illustrate the average OTUs observed (y-axis) from the sequences per sample (x-axis). From the curves we see that the number of sequences per sample leveled off for all the samples. This suggests that deeper or additional sequencing does not add significantly to the percentage of observed OTUs. It also indicates that differences in the number of sequences are not a significant contributor to the observed diversity in microbiota or the observed OTU in the samples. There are five samples (BF_F01, WB_P07, WB_F04, *Other*_F03, and *Other*_F05) that seem to be consistently higher than the rest, but after careful review of the materials, the samples were included in the analysis.
