# Supplementary material for: Microbial Diversity in Bushmeat Samples Recovered from the Serengeti Ecosystem in Tanzania
Source: Sci Rep. 2019 Dec 2;9:18086. doi: 10.1038/s41598-019-53969-7 (PMC6888819; doi:10.1038/s41598-019-53969-7)
Supplement: Supplementary file 3 — Supplementary Tables [file 41598_2019_53969_MOESM3_ESM.docx]

**Supplementary Tables:**

**Microbial Diversity in Bushmeat Samples Recovered from the Serengeti Ecosystem in Tanzania**

**Robab Katani^1,2^, Megan A. Schilling^2,3^, Beatus Lyimo^4^, Triza Tonui^5^, Isabella M. Cattadori^2,6^, Ernest Eblate^4,7^, Andimile Martin^4^, Anna B. Estes^2,4^, Teresia Buza^2^, Dennis Rentsch^8^, Karen W. Davenport^9^, Blake T. Hovde^9^, Samson Lyimo^4^, Lydia Munuo^4^, Francesca Stomeo^5^, Christian Tiambo^5^, Jessica Radzio-Basu^1,2^, Fausta Mosha^10^, Peter J. Hudson^1,2,7^, Joram J. Buza^4^ and Vivek Kapur^1,2,3,4*^**

1Applied Biological and Biosecurity Research Laboratory, Pennsylvania State University, University Park, Pennsylvania, USA;

2The Huck Institutes of the Life Sciences, Pennsylvania State University, University Park, Pennsylvania, USA;

3Department of Animal Science, Pennsylvania State University, University Park,

Pennsylvania, USA;

4Nelson Mandela African Institution of Science and Technology, Arusha, Tanzania; 5Biosciences eastern and central Africa-International Livestock Research Institute (BecA-ILRI) Hub, Nairobi, Kenya (Currently at the European Molecular Biology Laboratory (EMBL), Heidelberg, Germany);

6Department of Biology, Pennsylvania State University, University Park; ^7^Tanzania

Wildlife Research Institute, Arusha, Tanzania;

8Lincoln Park Zoo, Chicago, Illinois, USA;

9Los Alamos National Laboratory, Los Alamos, New Mexico, USA;

10Ministry of Health Community Development Gender Elderly and Children, Dar es Salaam, Tanzania.

**Supplementary Table 1.** Metadata of the 56 samples collected from West Serengeti region in Tanzania. Species include wildebeest (WB), buffalo (BF), and less prevalent species (*Other*). The “*Other*” species are as follows; Eland (EL), Gazelle (GZ), Giraffe (GR), Porcupine (PC), Rabbit (RB), Sheep (SH), Topi (TP), Warthog (WH), and Zebra (ZB). Each sample is shown by ID code corresponding to the figures, species, condition, season, and the district in Western Serengeti where they were collected from. "*Other*" species shown in column (ID1) are described in ID2 column.

**Supplementary Table 2.** Groupings of hierarchical clustering of the samples at phyla level including all variables (species, season, region, and condition) as shown in the Figure 5.

**Supplementary Table 3.** Variability in the abundance of the different bacterial taxa by species (Buffalo, *Other*, Wildebeest), condition (fresh and processed), season (dry and wet), and region (Bunda, Tarime, and Serengeti) at the 2 taxonomic levels. Significant PerMANOVA values (1000 permutations, threshold: p<0.05) with the Sum of Squares (SS) and degree of freedom (df) using the Bray-Curtis diversity matrix are reported in bold.

**Supplementary Table 4.** Elliptical distances between the centers of ellipses depicted in the microbial diversity PCoA of figures 4 and supplemental figure 5. At both phylum and family level, the largest distances are between Tarime and Serengeti, followed by Tarime and Bunda. At family level, the largest distance is between buffalo and wildebeest samples (bold values).

1
